# The Empowering Role of Mobile Apps in Behavior Change Interventions: The Gray Matters Randomized Controlled Trial

**DOI:** 10.2196/mhealth.4878

**Published:** 2016-08-02

**Authors:** Phillip J Hartin, Chris D Nugent, Sally I McClean, Ian Cleland, JoAnn T Tschanz, Christine J Clark, Maria C Norton

**Affiliations:** ^1^ Computer Science Research Institute Ulster University Newtownabbey United Kingdom; ^2^ Computer Science Research Institute Ulster University Coleraine United Kingdom; ^3^ Department of Psychology Utah State University Logan, UT United States; ^4^ Department of Family, Consumer, and Human Development Utah State University Logan, UT United States

**Keywords:** behavior, health behavior, behavior change, motivation, Alzheimer disease, smartphone

## Abstract

**Background:**

Health education and behavior change programs targeting specific risk factors have demonstrated their effectiveness in reducing the development of future diseases. Alzheimer disease (AD) shares many of the same risk factors, most of which can be addressed via behavior change. It is therefore theorized that a behavior change intervention targeting these risk factors would likely result in favorable rates of AD prevention.

**Objective:**

The objective of this study was to reduce the future risk of developing AD, while in the short term promoting vascular health, through behavior change.

**Methods:**

The study was an interventional randomized controlled trial consisting of subjects who were randomly assigned into either treatment (n=102) or control group (n=42). Outcome measures included various blood-based biomarkers, anthropometric measures, and behaviors related to AD risk. The treatment group was provided with a bespoke “Gray Matters” mobile phone app designed to encourage and facilitate behavior change. The app presented evidence-based educational material relating to AD risk and prevention strategies, facilitated self-reporting of behaviors across 6 behavioral domains, and presented feedback on the user’s performance, calculated from reported behaviors against recommended guidelines.

**Results:**

This paper explores the rationale for a mobile phone–led intervention and details the app’s effect on behavior change and subsequent clinical outcomes. Via the app, the average participant submitted 7.3 (SD 3.2) behavioral logs/day (n=122,719). Analysis of these logs against primary outcome measures revealed that participants who improved their high-density lipoprotein cholesterol levels during the study duration answered a statistically significant higher number of questions per day (mean 8.30, SD 2.29) than those with no improvement (mean 6.52, SD 3.612), *t*_97.74_=−3.051, *P*=.003. Participants who decreased their body mass index (BMI) performed significantly better in attaining their recommended daily goals (mean 56.21 SD 30.4%) than those who increased their BMI (mean 40.12 SD 29.1%), *t*_80_ = −2.449, *P*=.017. In total, 69.2% (n=18) of those who achieved a mean performance percentage of 60% or higher, across all domains, reduced their BMI during the study, whereas 60.7% (n=34) who did not, increased their BMI. One-way analysis of variance of systolic blood pressure category changes showed a significant correlation between reported efforts to reduce stress and category change as a whole, *P*=.035. An exit survey highlighted that respondents (n=83) reported that the app motivated them to perform physical activity (85.4%) and make healthier food choices (87.5%).

**Conclusions:**

In this study, the ubiquitous nature of the mobile phone excelled as a delivery platform for the intervention, enabling the dissemination of educational intervention material while simultaneously monitoring and encouraging positive behavior change, resulting in desirable clinical effects. Sustained effort to maintain the achieved behaviors is expected to mitigate future AD risk.

**Trial Registration:**

ClinicalTrails.gov NCT02290912; https://clinicaltrials.gov/ct2/show/NCT02290912 (Archived by WebCite at http://www.webcitation.org/6ictUEwnm)

## Introduction

### Health Education Programs and Alzheimer Disease

Health education programs have demonstrated their effectiveness in educating individuals with targeted knowledge relating to risk factors of various diseases [1,2]. With this knowledge, individuals are subsequently capable of making educated decisions regarding lifestyle choices, which may have a significant effect on their future health outcomes. Most health education programs target the leading causes of mortality [3], such as heart disease and stroke [4], cancer [5], diabetes [4], and respiratory diseases [3,6]. Nevertheless, only a limited number of studies have been conducted with a focus on health education for Alzheimer disease (AD) risk reduction, despite being the sixth leading cause of total mortality in the United States [7] and the first and second leading cause of mortality of females and males older than 80 years, respectively, in the United Kingdom [8].

### Alzheimer’s Disease Risk

Unfortunately, efforts to create a vaccine for AD have proven unsuccessful. Nevertheless, findings from clinical and epidemiological studies have suggested that behavioral, social, and environmental factors may delay or prevent the onset of AD [9,10]. At the G8 dementia summit held in December 2013, clinical AD experts called upon the governments of G8 countries to make the prevention of AD a major health aim, while highlighting the suggestion to study the risk factors associated with the disease [11]. Currently, identified risk factors include dietary factors (food choices; body mass index, BMI; endocrine disorders; and diabetes) [12], cardiovascular factors (sedentary lifestyle, hypertension, and high cholesterol) [13], and psychosocial factors (education, higher work complexity, social participation, and intellectual activities). Importantly, these factors are modifiable and therefore have the potential to be useful targets for the prevention of cognitive decline and AD through behavioral change programs.

The health education interventions that individually targeted such factors for other conditions exhibited positive results, suggesting that a similar effort targeting AD would be likely to result in the desirable adoption of healthy behaviors [14]. Given that many of the risk factors can be interdependent (eg, BMI and sedentary lifestyle), a multifactorial preventative intervention targeting several risk factors simultaneously presents the greatest likelihood of being effective [9]. A study by Lin et al simulated the potential health and economic effects of addressing AD risk factors. Their simulated scenarios found that as each of the factors were addressed, additional unintended benefits were observed, such as lowering the risk of other chronic diseases (diabetes, heart disease, and stroke), accompanied with a 10% reduction in BMI in those who were overweight [15].

It is therefore hypothesized that a health education program that provides evidence-based information regarding AD risk factors and prevention methods may have the additional benefit of reducing risk for other health conditions, with particular improvement in cardiovascular functions.

### When to Intervene?

Although the risk factors for cognitive decline and AD have been identified and are natural targets for a behavior change intervention, there is variation in the literature as to when such interventions should take place. It has become widely accepted that the neuropathological processes involved in AD begin decades before symptoms emerge [16]; however, behavior intervention programs relating to AD have focused almost exclusively on an elderly population (65-80 years) [17], rather than introducing interventions in midlife (40-64 years). Numerous midlife health markers have been linked with higher late-life AD risk, such as obesity [18], hypertension [19], serum cholesterol levels [20], and physical activity during leisure time [21], all of which can be addressed simultaneously via a behavior change intervention to prevent the onset of the disease.

It is therefore further hypothesized that an intervention targeted at those in midlife holds the greatest potential for reducing future risk of developing AD.

### Technology as an Intervention Tool

To appropriately distribute an education-based behavioral intervention program, a suitable method of delivery is required. This paper will describe the numerous empowering roles, for both end users and investigators, that the mobile phone has facilitated during an evidence-based multi-domain behavior change intervention, entitled “The Gray Matters study,” which aims to reduce the future risk of developing AD, while in the short term promoting cardiovascular health (ClinicalTrials.gov identifier: NCT02290912) [22].

### Background

This section details related works in the areas of behavior change interventions and public education programs, covering how intervention material is typically delivered to users, how engagement is maintained, and how various behaviors are tracked. This study also investigated the current use and potential unexplored capabilities of technology in each of these areas.

#### Delivery of Interventions

The term *delivery* encompasses both the psychological message of the intervention material and also the mode of distribution. Educational material for the purpose of behavior change can be designed to evoke certain emotional responses. Fear appeals, that is, the use of persuasive messages to stimulate fear based on harmful outcomes that are associated with dangerous lifestyle practices, have been used extensively for more than 60 years [23,24]. Perhaps not surprisingly, they have been found to be rather ineffective and can produce a polarizing effect within the intended cohort [24]. Because most people wish to think of themselves as healthy, such threatening information can lead to a defensive response, motivating intended recipients to avoid exposure to the material in the future [24]. An example of a public health campaign aiming to use fear appeal can be observed globally in tobacco packaging via the use of clear warning messages and graphic images of smoking-related diseases.

**Figure 1:**
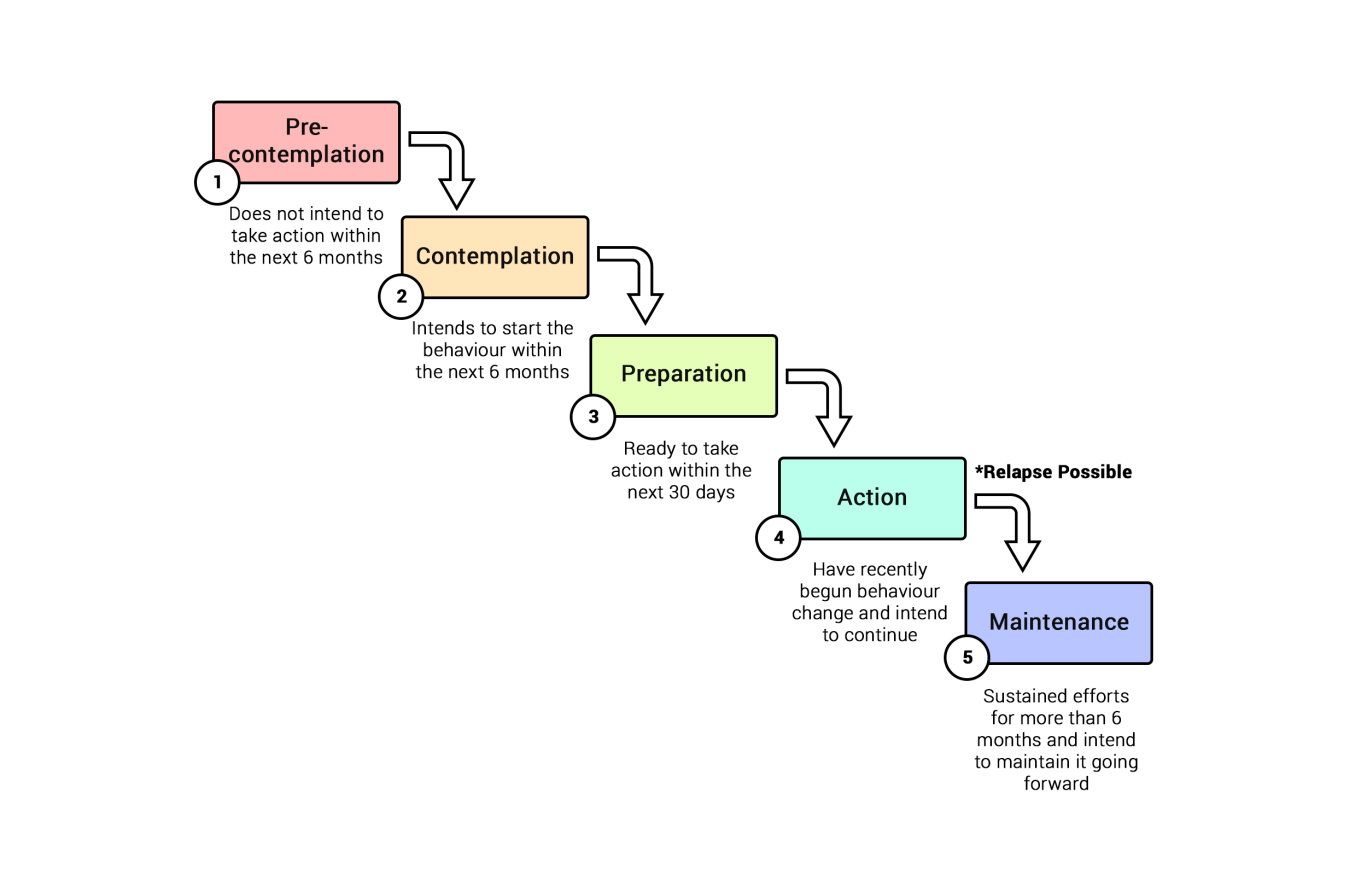
Stages of behavior change within the Transtheoretical Model.

There are a number of ways in which an individual's behavior change can be theoretically modeled [25]. These include the widely cited and applied Theory of Planned Behavior (TPB) [26], the Health Belief Model (HBM) [27,28], and the Transtheoretical Model (TTM). The most apt models for planning a behavior change intervention, however, are the HBM and the TTM. The TPB is useful in predicting certain behaviors, and for retrospective analysis, but is not considered useful or effective in relation to designing and planning an intervention that should result in behavior change [29]. The aforementioned example utilizing fear appeals, including cues to action and perceived threats, belongs to the HBM. The TTM, however, is a stage theory that is often used as a guiding framework for many health-related interventions. This model posits that an individual’s willingness to make behavioral changes is driven by his or her readiness to change. Stages of readiness are described as precontemplation, contemplation, preparation, action, and maintenance, with relapse to prior unhealthy behaviors possible between the action and maintenance stages (Figure 1) [30].

This type of approach aims to empower an individual via education and introspection to create a positive feedback loop as behaviors are changed over time [31]. For AD prevention, this TTM approach would be performed by educating an individual on specific behaviors, having the individual introspectively assess his or her own behaviors, and setting attainable future goals to target change. On completion of these goals, the individuals are affirmed of their efforts and subsequently reevaluate their goals, thus creating a positive feedback loop.

Using such an approach in a health care intervention would rely heavily on the ability to personalize the education material and the ability to set attainable goals. To achieve such an intervention in the physical world, using people, buildings, and paper, would require enormous resources and planning. Fortunately, Internet-based technologies can reduce this burden, by digitally delivering intervention material.

Mobile and Internet-based technologies have been accepted as suitable and sustainable methods to deliver intervention material in various studies. Mobile phone–based delivery, such as short message service (SMS), has been used extensively and successfully in the literature to support portable and widespread interventions [32]. Internet-based services, such as email and website portals, have also been used extensively and with good success [33]. The ability to digitally disseminate material offers many advantages to health care investigators and end users alike: notably, personalization of material, increased scalability, and reduced expenses.

#### Maintaining Engagement

Evidence from Internet-based interventions suggests that repeated visits are necessary to achieve sustainable change [33]. Nevertheless, visitor engagement with these interventions is typically lower than expected, with many users opting out before becoming fully exposed to all the intervention material, resulting in suboptimal outcomes [33]. There is therefore the need to encourage and maintain engagement with interventions, while enhancing an individual’s motivations to return at a later date.

Gamification is the application of game design techniques and mechanics to nongaming domains [34]. To encourage engagement within a game, game designers utilize mechanics such as points, level systems, avatars, badges, and leaderboards. These reward systems encourage continual progression, with the ultimate aim of maintaining engagement. Recently, there has been a surge of interest in the use of gamification for behavior change studies, given that these reward systems help to promote engagement. Young adults and children are especially attracted to games, with virtually all young children having access to gaming consoles, computers, and mobile phone games [35]. As such, gamification elements have been used to educate and encourage desirable behaviors in children, such as increasing intake of fruits and vegetables through the use of fictional avatars [36], preventative education on substance abuse and risk using mobile phone and tablet apps [37], and an obesity prevention intervention via mobile and Web platforms [38]. The use of gamification in adult behavior change studies, however, is limited.

For health-conscious adults, commercially available mobile phone apps and activity tracker companies, such as Strava, Fitbit, and Nike, use gamification elements extensively in their efforts to maintain and promote continual engagement. Although each platform has its own approach, they all record health-related data; examples include monitoring physical activity levels, tracking meals, and monitoring sleep quality. From these data, various performance metrics are calculated from which achievements are rewarded, such as badges and trophies. In addition, a user can view, typically at a high level via interactive graphs, their performances across time, allowing them to become informed of their behaviors and their resulting outcomes. Social sharing of recorded data also plays a role in enabling gamification elements, such as leaderboards, allowing users to compare their efforts with those of others. Apple and Google, whose mobile phone platforms combined account for 96.3% of the worldwide market share [39], are now shipped with iOS HealthKit and Google Fit services preinstalled. The aforementioned services are proprietary to their platforms; however, they act to consolidate the available data of various health-related apps and activity trackers into one common interface. The inclusion of such services into the base functionality of the most extensively adopted mobile phone platforms in the world shows the market’s anticipation of widespread adoption of health-related apps.

It is therefore hypothesized that the combination of constantly accessible, highly interactive, and individually tailored feedback, combined with gamification elements, such as rewards and leaderboards, would have the largest opportunity to maintain and encourage engagement with adults in a behavior change study [40], given the advantages that each element brings.

#### Reporting Behaviors

To accurately assess the effect of a behavior change intervention, the validity of the reported behaviors must be accurate. There are numerous methods by which behaviors can be recorded within an intervention, including diaries, questionnaires, direct observation, and by proxy reports [41].

Diaries present a low-cost, easily maintained, and time-efficient method of recording behaviors; however, they are open to cognitive bias due to subjective self-assessment and rely heavily on the person’s ability to accurately recall past events [42].

Direct observation offers health investigators an accurate portrayal of behaviors within the given window of observation [43]. They are believed to offer more truthful recordings and can be used as a method to increase precision and accuracy for the purposes of validating self-reported behaviors. With regard to monitoring physical behaviors, total energy expenditure can be calculated using calorimetry (ie, doubly labeled water), heart rate monitors, and motion sensors [43]. Although such approaches offer exceptional accuracy, they are intrusive, expensive, and time intensive. It is also the case that the Hawthorne effect, commonly referred to as the observer effect, may change how an individual behaves under direct observation, and observations made may not be a true reflection of their behaviors outside of the observation window [44].

Self-reported questionnaires are commonly used in large-scale longitudinal studies because of their uniformity in questioning, repeatability, and ability to extract qualitative and quantitative information [45]. Quantitative oriented questionnaires, seeking to gather quantifiable information about past events, such as the “number of glasses of water consumed today,” can be at risk of cognitive bias and recall inaccuracy. Nevertheless, a comparative study seeking to validate previous day recall accuracy for active and sedentary behaviors when compared with direct observation found agreement of 85% or higher in certain conditions and suggests adults can accurately report their behaviors using previous day recall [46].

Proxy reporting is typically used when the subject in examination is somehow dependent on another adult, such as young children and the elderly. A study assessing the level of agreement between 6425 children and their parents regarding dietary, physical, and sedentary behaviors reported a mean agreement rate of 43% [47]. Similarly, studies assessing memory recall for the same events in children, young adults, and the elderly showed that the reports of the elderly were as complete as the children’s but were the least accurate overall [48]. This highlights both the potential inaccuracies of self-reporting within certain cohorts and the need for ground-truth data due to the rate of disagreement found in reporting utilizing a proxy.

Although a variety of approaches can be employed to record behaviors, each has its own distinctive weaknesses relating to accuracy, repeatability, scalability, and cost [43]. There is a need for an objective mediator to draw agreement across the various approaches. Pervasive computing may provide such a solution.

The widespread public adoption of mobile phones, smartwatches, and wearable technology has enabled computing to become truly pervasive. Wireless digital devices can enable the digitization of individuals’ behaviors, often without the need for interaction. Wearable wrist-worn devices can be used to calculate an individual’s energy expenditure and step count [49], current activity [50], sleep quality [51], and heart rate [52]. Mobile phones, via the use of onboard accelerometers and the Global Positioning System, can also track physical activity levels [53] and sleep efforts [54], while various apps encourage self-reporting of food consumption [53], enabling immediate calculation of calorie consumption. In addition, social media websites contain a plethora of social interactions that can be analyzed for behavioral trends [55]. There is an abundance of potential use cases for such technology in the self-management of one’s health, yet the adoption of this technology for the purpose of public health education or behavioral change interventions is extremely limited. Eric Topol, a physician who has been heavily involved with wireless medicine since its inception, states in his book: “Our health care approach is reactive, and, as a result, we have a world of chronic diseases, most of which are poorly managed, such as congestive heart failure, high blood pressure, and diabetes, or not managed at all, as in the case of Alzheimer’s.” He continues, “Now comes a new wave of technology to not only improve the outlook for the chronic diseases of today but shift the capability, for the first time, to true prevention” [56].

To leverage this opportunity, the Gray Matters study has designed a clinically focused, technology-driven intervention program. An interdisciplinary team of computer scientists, biomedical engineers, mathematicians, psychologists, gerontologists, epidemiologists, and statisticians designed the Gray Matters mobile phone app: an app intended to deliver health education material, promote and monitor behavior change, and encourage the motivations of the participants via gamification elements [57].

## Methods

### Topics Addressed

This section details the study design and the technical development of the Gray Matters app, including study components, participant recruitment, eligibility criteria, outcome measures, and procedures.

### Study Design

The study was a randomized controlled trial (RCT) consisting of 144 subjects who were randomly assigned to either treatment or control group. The treatment group was not given a strict regimen and therefore a wide range of engagement levels were anticipated. A uniform random number generator (0,1) within SPSS v21 was used to randomize participants to treatment and control groups, with the aim of allocating 1/3 to control and 2/3 to treatment. The rationale for a 2:1 ratio for treatment and control was in consideration of the full autonomy given to each participant in the study. On recruitment, each participant was asked which behavioral domain or domains were of greatest interest to him or her to improve upon. In order to have a reasonably good power to study both the change in individual behavioral domains and its effects on those who wished to improve on particular domains, the ratio was adjusted to accommodate this. The intervention was delivered over a 6-month period, commencing in April 2014, with posttest collection performed at the close of the trial.

### Recruitment

Recruitment of participants was achieved by emailing announcements to faculty, alumni, and staff of Utah State University and distributing flyers at health fairs and other venues, assisted by the local health department and their community liaisons. For those interested a prescreening eligibility survey was completed. Eligibility criteria included (1) age between 40 and 64 years, (2) BMI no higher than 41, (3) possession of a mobile phone or tablet (iOS or Android), (4) fluency in the English language, (5) residence in Cache County, and (6) not having any of the following medical conditions: pregnancy, dementia, unmanaged diabetes, or untreated major depression.

### Statistical Power

To achieve 80% statistical power to detect a medium effect size (Cohen’s *d*=0.50) when comparing the difference between 2 independent means at a 2:1 (treatment to control) ratio, 96 treatment and 48 control (144 total) participants were needed, calculated using G*Power [58]. Upon randomization, 104 participants were assigned to treatment and 42 to control. To avoid intracouple contamination of intervention material, married couples were assigned to the same randomized group (n=12).

### Outcome Measurements

Primary outcome measures of the trial registration included a set of anthropometric measures, blood-based biomarkers, objective cognitive testing, and behavior in targeted domains. Secondary outcome measures included metacognition, motivation, readiness for change, sleep quality, social engagement, depression, and couple satisfaction (among married persons). Tables containing full summaries of all recorded values at the beginning of the study, for all 146 Gray Matters study participants, can be found in the study by Norton et al [22]. In addition to the outcome measures recorded as part of the trial registration, app usage metrics and behavioral data collected through self-reporting within the app are also analyzed [57].

### App Design, Development, and Deployment

This subsection details the design of the Gray Matters mobile phone app and accompanying educational material, the development of the systems to support the collection of behavior data, and the method of deployment to the cohort within the Gray Matters study.

#### Educational Material

To enable the dissemination of evidence-based educational material relating to AD risk and prevention strategies, more than 130 peer-reviewed journals and papers relating to AD risk were analyzed. From the analysis, it was identified that risk factors and their prevention methods could be categorized into 6 domains: food, physical, cognitive, social, sleep, and stress. For these 6 domains, fact and suggestion pairs were produced (hereafter referred to as daily facts). An example daily fact from the food domain is as follows: “Consuming high amounts of processed foods is related to cognitive decline”; “Try a fresh salad for dinner instead of something from a box”. In total 164 succinct daily facts were produced across the 6 domains: physical (23), food (66), social (27), sleep (14), cognitive (24), and stress (10).

In addition to the daily facts, questions were designed for each domain to capture behaviors relevant to AD risk. All questions were quantitative in nature; however, they contained a mixture of subjective and objective questions. For example, a user may be asked to report the number of fruits and vegetables they consumed in a day (objective) and also rate their quality of sleep on a scale of 0-10 (subjective). In addition to the questions for the original 6 behavioral domains, a question was added to collect the activity data observed via a wearable device. In total 12 questions were designed for the domains: physical (2), food (3), social (1), sleep (1), cognitive (2), stress (2), and wearable activity monitor (1). For each question, a recommended value was extracted from external sources, such as the World Health Organization, the American Heart Association, the National Institutes of Health, and the Centers for Disease Control and Prevention’s (CDC) recommended daily targets (see Table 1). The recommended value served two purposes: to act as an observable goal for the participant and as a means by which a participant’s performance could be calculated, relative to other participants.

**Table 1:** The questions presented to the user, showing their minimum, maximum, and recommended values.

Domain	ID^a^	Question	Min^b^	Max^c^	Recommended (source)	Type
Cognitive	1	How many minutes did you spend today doing “novel mental exercises”?	0	120	30 minutes (NIH^d^)	Objective
Cognitive	2	How many minutes did you spend today doing “cognitively stimulating activities”?	0	120	30 minutes (NIH)	Objective
Food	3	How many cups of fruits and vegetables did you eat today?	0	10	5 cups (CDC^e^)	Objective
Food	4	How many ounces of whole grains did you eat today?	0	10	3 ounces (CDC)	Objective
Food	5	How many servings of nuts, seeds, or legumes did you eat today?	0	5	1 serving (CDC)	Objective
Physical	6	How many minutes of “moderate” physical activity did you do today?	0	60	30 minutes (AHA^f^)	Objective
Physical	7	How many minutes of “vigorous” physical activity did you do today?	0	60	20 minutes (AHA)	Objective
Sleep	8	How would you rate your sleep promotion efforts over the past 24 hours?	0	5	5	Subjective
Social	9	How would you rate your social engagement in the last 24 hours?	0	7	7	Subjective
Stress	10	How much effort have you put into decreasing your stress over the past 24 hours?	0	10	10	Subjective
Stress	11	On a scale of 1-10 how would you rate your stress level over the past 24 hours?	1	10	1	Subjective
Wearable	12	How many Nike Fuelpoints did you earn today?	0	5000	2000 (Nike)	Objective

^a^ ID: identification.

^b^ Min: minimum.

^c^ Max: maximum.

^d^ NIH: National Institutes of Health.

^e^ CDC: Centers for Disease Control and Prevention.

^f^ AHA: American Heart Association.

#### App Development

The mobile phone app was developed natively for both Apple iOS and Google Android mobile phones. The decision to develop for both platforms was made based on rudimentary market analysis of mobile phone sales within the intended cohort’s location (Cache County, Utah, USA). Initially the app was developed for iOS 7.x devices, including iPhone and iPad, as the analysis showed a favoring for these devices in the area. As technology screening during the recruitment phase progressed, additional demand appeared for an Android version, which was subsequently developed. The functionality and visual layout of both versions are virtually indistinguishable, yet allowing enough flexibility to adhere to each platform's user interface design guidelines [59,60].

As the primary method to deliver health education material and track behavior change in the study, the app was designed to fulfill the following core functions:

1. Presentation of educational material relating to AD risk and prevention strategies.

2. Facilitation and recording of behavior self-reporting.

3. Calculation and presentation of personalized feedback based on reported behaviors.

#### User Interface Structure

Each function was presented in the user interface as a tab in the aforementioned order, allowing for easy and logical navigation. For the end user, the functions are displayed as the Tips tab, Log tab, and Performance tab.

##### Tips

**Figure 2:**
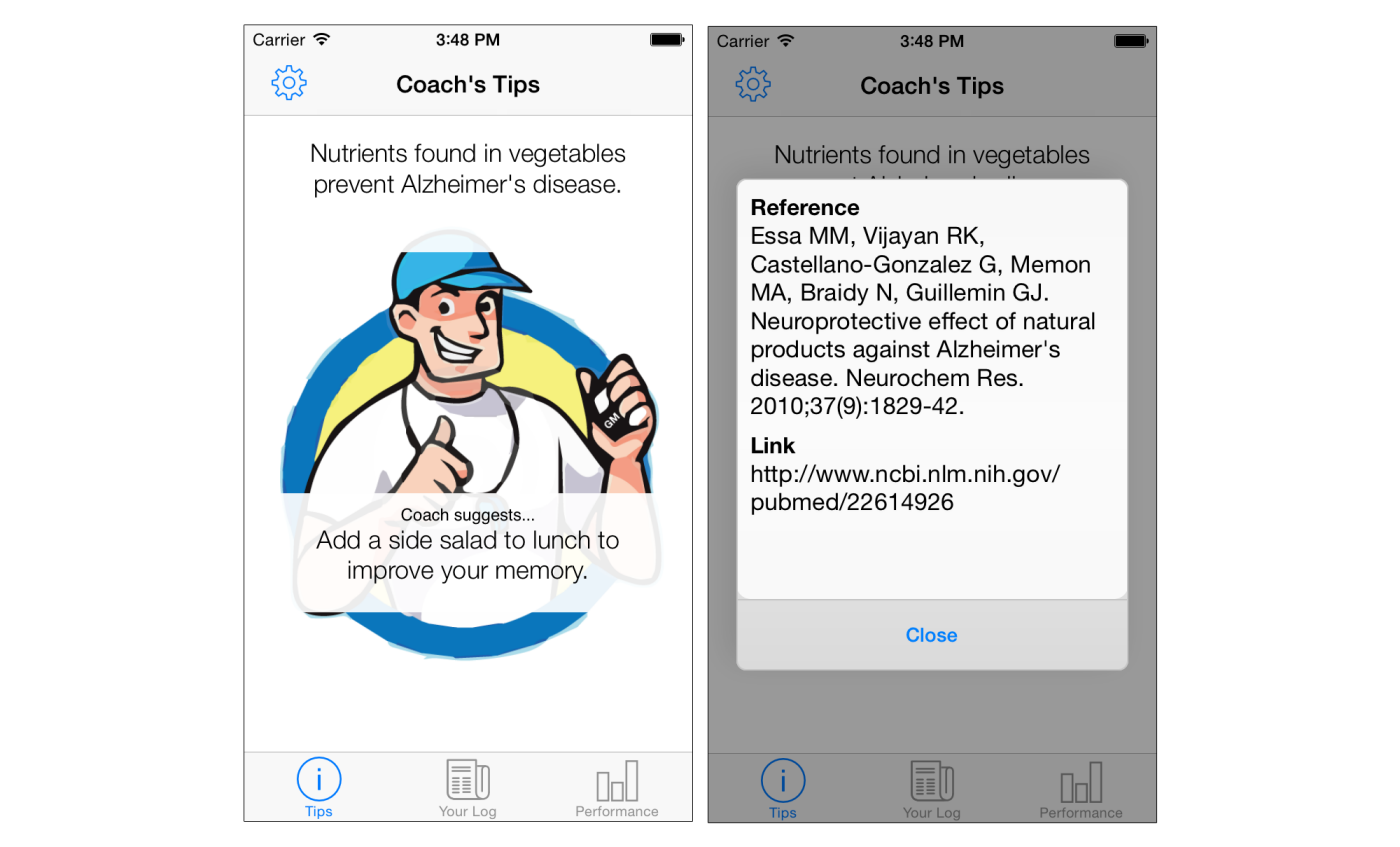
Tips tab main screen showing daily fact (fact and suggestion pair; left). Evidence-based literature reference and link are displayed when the fact section of the daily fact is tapped (right).

This tab displays the evidence-based daily facts regarding risk factors and preventative strategies. The tab also contains a sports coach avatar, designed to aid visual delivery and personification of the recommendations offered in the daily fact (refer to Figure 2). The text is also tappable, which presents a pop-up box displaying the reference source and a URL, which can be navigated to if further information is desired. Although it may be argued that for the layman the use of clinical references may be deemed superfluous, its inclusion considers the broad spectrum of potential users’ needs and also acts to instill confidence in the recommendations provided.

##### Log

**Figure 3:**
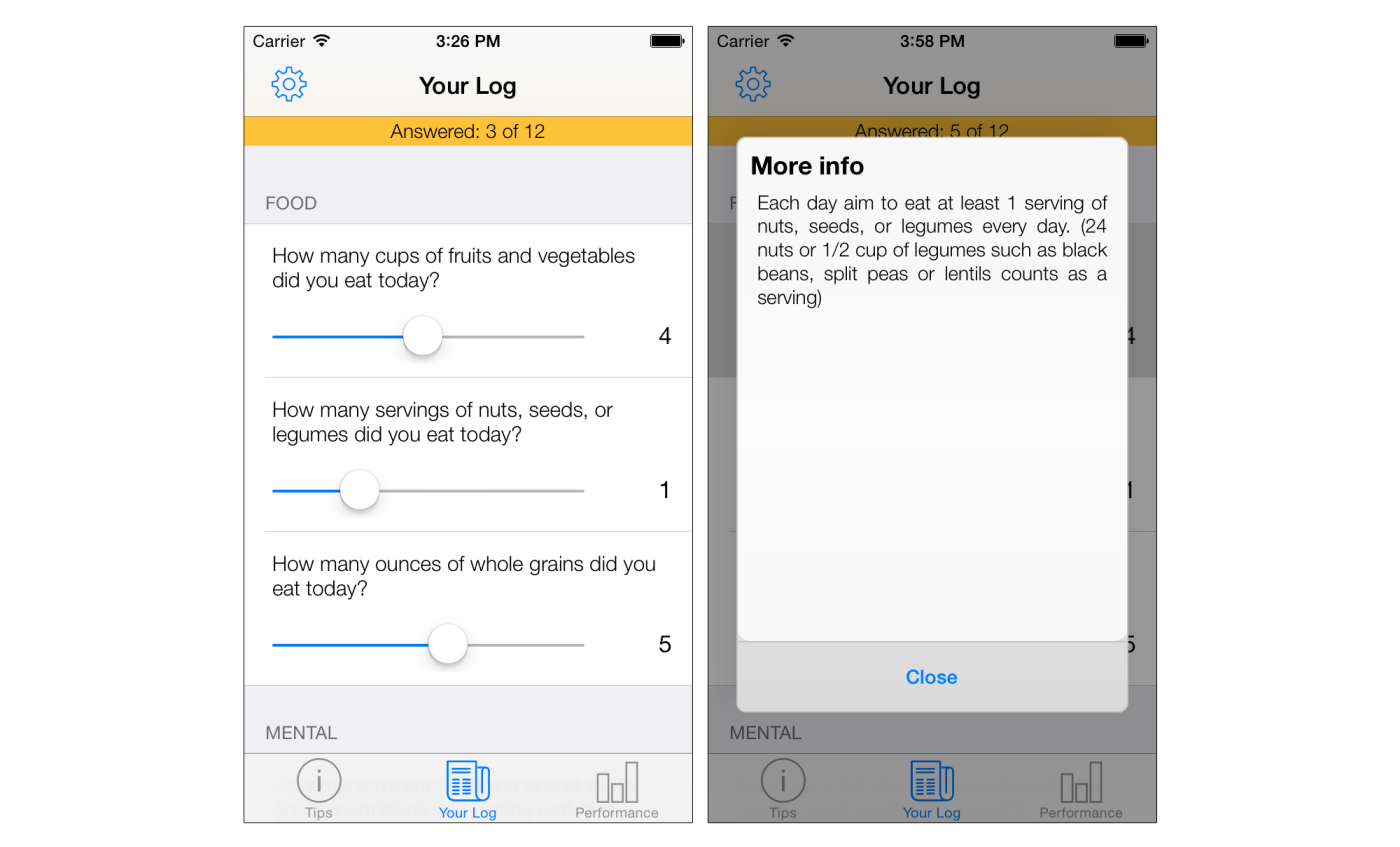
Log tab main screen showing list of questions ordered by domain (left). The sliders can be dragged to adjust logged amount. Dialog box containing additional information displayed for a question regarding servings of nuts, seeds, and legumes (right).

This tab facilitates the collection of behavioral data via self-reporting. As seen in Table 1, a total of 12 questions were designed to collect all relevant behavioral data for the study. During the requirements elicitation, it was specified that data entry for all questions should take no more than 2 minutes to complete, to reduce time burden for the study participants. As such, a time-efficient approach was developed. The questions are presented in a list, ordered by their domain (refer to Figure 3). Data entry is achieved by moving a fixed-width slider across the screen until the desired value is presented. As the questions were designed to be quantitative, the use of a slider allows data from the questions to be represented in a scale, using increments of 1. In most instances, for objective data types, the upper limit of the slider is twice the recommended value, facilitating those who wish to overachieve. The use of a recommended value is an observable target for the user, which when achieved acts to reward and reinforce the desired behaviors, as outlined in the action stage of the TTM [30].

In order to reduce subjectivity in the questions, a user who is unsure about the exact meaning of the question may tap on it to present an expanded and elaborated phrasing of the question, including examples. For example, the question “How many minutes of moderate physical activity did you do today?” may be considered subjective if the term “moderate” is not understood. To counter this, tapping on the question presents the description “The CDC recommends 2 hours 30 minutes of ‘moderate’ activity per week. Examples of moderate activity are walking, skiing, raking leaves, washing the car.”

By answering each question, the users can longitudinally track their behaviors across all 6 domains, including their wearable device metrics. Answering all 12 questions is not compulsory; however, it is advantageous for both the participants and the study investigators as it increases the granularity of the data for each user and the study cohort as a whole. Answers are uploaded to a remote server via http protocols, using the open-standard JSON format to package the data.

##### Performance

**Figure 4:**
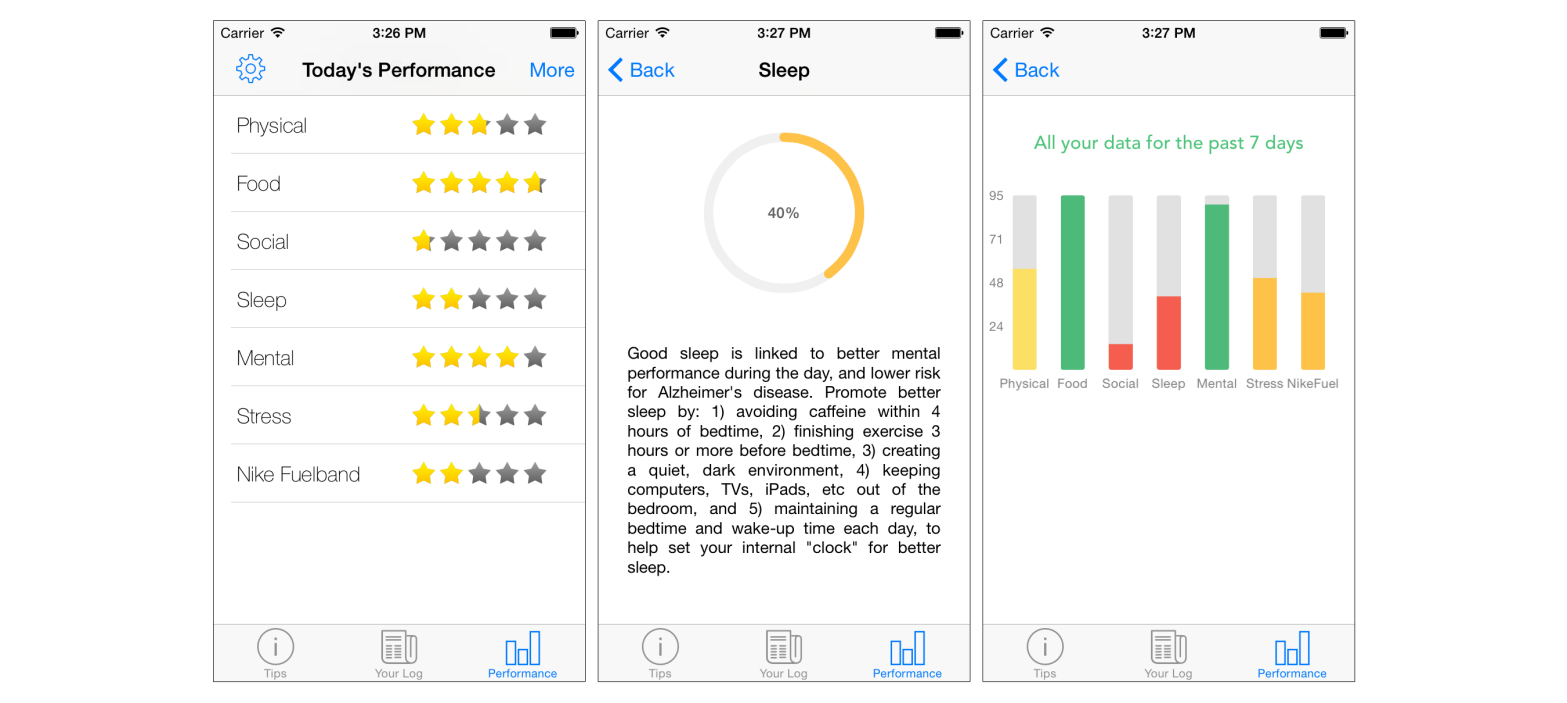
Performance tab showing star ratings for each domain, calculated by assessing a user’s reported values against recommended values (left). Expanded view of performance with a domain, containing additional information and helpful tips (center). A bar chart showing aggregated performance, as a percentage, from the previous week’s data (right).

**Figure 5:**
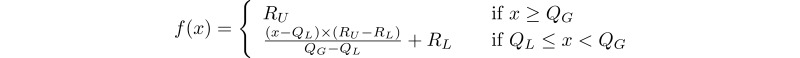
Equation developed by the author to normalize users performance metrics to a 5 star rating for visual representation.

The performance tab is designed to present various summaries of the data collected from the log tab, while encouraging continual participation via rewards. The main mode of presentation is via star ratings (refer to Figure 4). These stars can be considered a variant of points-earning system, a system commonly used to encourage continual progression within behavior change programs [61,62]. Utilizing the concepts explored in gamification, a user can achieve a maximum of 5 stars for each behavioral domain each day. This is achieved by reaching the recommended value, for each question, in each topic. As such, all recorded values in the logs must be normalized to within a range of 0-5 in relation to the recommended value. To perform this necessary step, the authors developed the equation presented in Figure 5.

In the equation, *x* is the user’s answer value to a particular question, *Q*_G_ is the goal value for the question, *Q*_L_ is the lowest possible value for that question, *R*_U_ is the upper boundary of the normalized result, and *R*_L_ is the lowest boundary.

The stars are designed to encourage and reinforce a participant’s effort to change his or her behavior. Because all domains can be viewed on screen at the same time, it provides a fast method to deliver visual feedback on the domains that require more effort and those that are under control. Users may also tap on each domain to receive additional pertinent information and an additional graphical representation of their efforts. The users may also view their performance aggregated across the previous 7 days in the form of a spider diagram or a bar chart. Again, this serves to visually assist the participants in understanding their behaviors for the purpose of self-affirmation.

#### Remote Monitoring

Participant data from the app are uploaded to a remote MySQL server located at Ulster University. This occurs in real time if a user has a valid Internet connection, via Wi-Fi or mobile network. This instant transmission of behavioral data offers health investigators in the study an opportunity to perform immediate analysis, at any given point during the intervention. Because the data are in a structured digital format, very little human processing or interaction is required to run queries or statistical analysis. This presents a huge advantage over studies that control their data collection and processing via paper-based postal services and questionnaires [63].

#### App Analytics

To exploit the opportunity and increase the granularity of available data, the app also monitors all in-app actions using proprietary and open-source analytical tools. These analytical data enable the investigators to examine the profile of the average user and provide insight into how the app is actually being used. Examination of the analytical tracking data also highlights features that fulfill their purpose, while also identifying problematic areas of the app, flagging them to be addressed in future updates. Components of the app that contain analytical tracking code include app launching, tab navigation, updating log values, changing notification times, question detail expansion, and performance analysis.

### Additional Intervention Components

In addition to the aforementioned mobile phone app, participants in the treatment group had access to a number of components to encourage behavior change. These included a wrist-worn activity monitor, booster events, a personal coach, and a study website.

#### Wrist-Worn Activity Monitor

Each participant was given a Nike FuelBand SE activity monitor. This device is worn on the wrist and serves to collect information such as steps taken, stairs climbed, and minutes of activity. This information is then consolidated into Nike’s proprietary metric of “NikeFuel points.” This device not only serves to collect data, but also acts as a physical reminder and motivator to increase levels of activity. Participants were asked to manually enter their total number of NikeFuel points earned at the end of each day via the mobile phone’s log tab.

#### Booster Events

All participants had the option of attending organized booster events. Each booster event was designed to emphasize the link between a specific domain and the risk it posed to developing AD, accompanied by preventative measures that the participants could apply in their daily lives. For example, a booster event that focused on the food domain hosted cooking classes that promoted sustainable healthy eating choices, while educating attendees about the link between the ingredients and AD risk. In total 46 booster events were organized and delivered across the 6-month intervention period.

#### Personal Coach

Participants also had access to a personal coach whom they could contact if they required assistance with any aspect of the behavioral domains. A team of 28 student interns with majors in the 6 behavioral domains volunteered to be personal coaches. Student coaches were trained in motivational interviewing and the TTM and provided a weekly email or text message exchange with their assigned participants to provide emotional support and encouragement for lifestyle change goals.

#### Study Website

Participants also had access to a password-protected website [64,65] that provided content for the 6 domains, support material for the use of the study technology, including instructional YouTube videos showing users how to install and use the app for iOS and Android. In addition, an email address was provided should additional issues arise.

### Exit Survey

An exit survey was designed to capture opinions of participants in the treatment group. The survey asked questions about app usage, motivations, their perceived behavior change, and social network usage. At the end of the study, 102 of the 104 participants completed this survey.

## Results

### A Brief Overview of This Section

This section presents the results from the RCT, including analysis of the treatments group’s adoption, typical usage, and perceptions of the app. This section also examines the app’s observed effects within the clinical and behavioral domains.

### App Adoption and Usage

**Figure 6:**
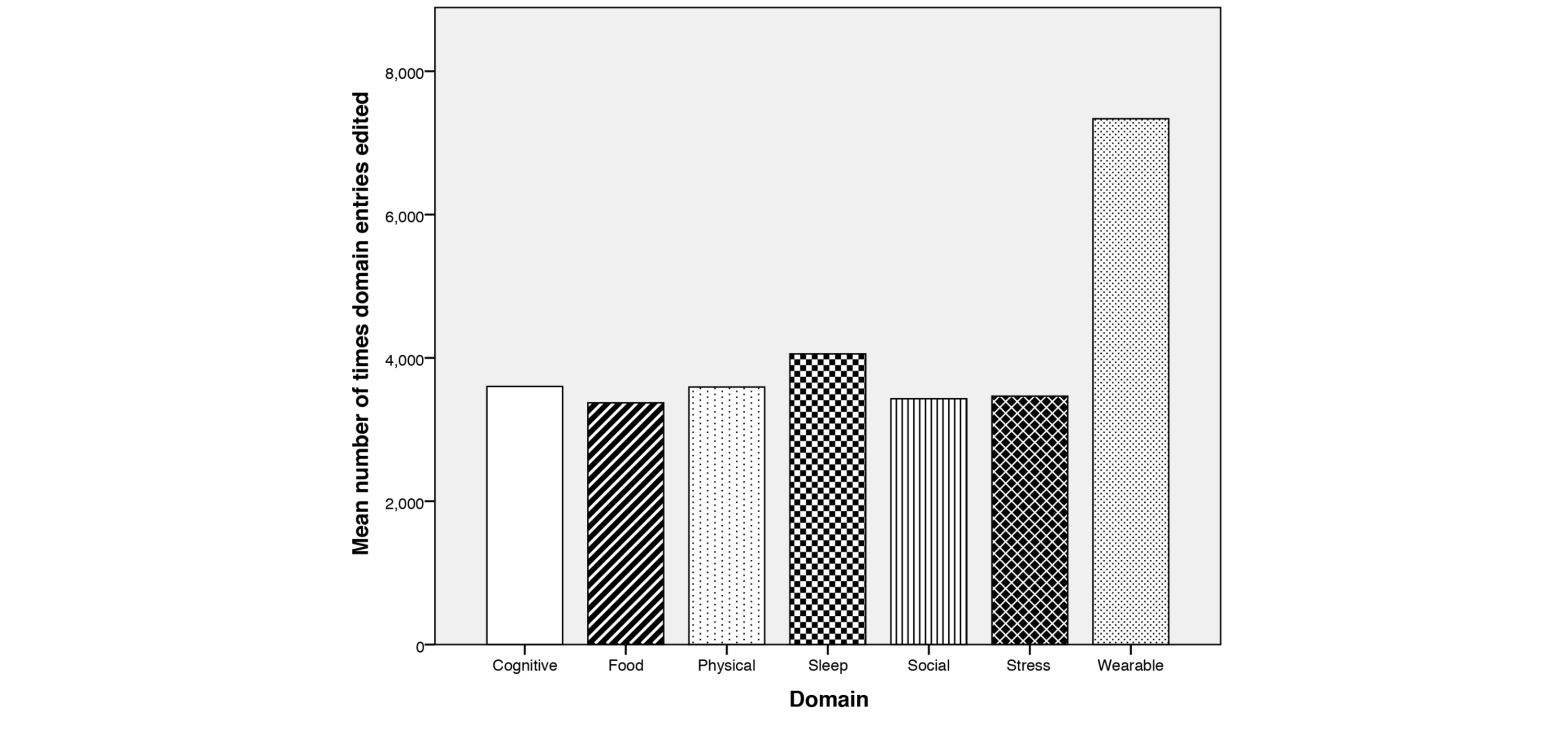
Bar graph showing the mean number of times each domain’s questions were edited using the sliders in the log screen, using updated analytical code, from week 18 to study end. The wearable domain is updated almost twice as often as the other domains.

In week 1 (April 10, 2014), the first iOS version of the app was released to the treatment group. This was performed through a launch event, in which attending participants were instructed how to sign up and download the app through the TestFlight platform. TestFlight is a platform by which developers can distribute apps to internal or external testers. This platform allowed the investigators to control visibility in the app marketplace, ensuring that only enrolled participants could see and install the app. By the end of week 1, a total of 31.7% (33/104) participants had installed the app on their iPhone and/or iPad. In week 3 (May 13, 2014), the first Android version of the app was released to the treatment group because of demand from Android users. Two weeks after this release, 19.2% (20/104) participants had installed the first version of the Android app. By week 10, a total of 86.5% (90/104) of participants from the treatment group had installed an iOS or Android version of the app on their mobile phone and/or tablet, with the remainder shortly afterward. Many users opted to install the Gray Matters app on both their mobile phone and tablet. Of the 104 users using the app, at the end of the study, 75.97% of all Gray Matters app sessions were on iOS devices (iPhone: 54.7%; iPad: 21.27%) and the remainder on Android devices (24.03%). Regarding self-reporting of behaviors, the average user answered 7.3 (standard deviation, SD 3.16) questions per day during their participation in the study. The average duration of each session with the app, across all devices, was 1 minute 55 seconds. This time is less than the originally specified goal of 2 minutes for a user’s session duration. For further information on app usage statistics during the initial 10 weeks of the study please refer to the study by Hartin et al [57]. Additional analytical tracking code was added to the app in week 18 to analyze the specific behaviors when answering questions in the log screen. The tracking code recorded the number of times the users altered their behavioral values (Figure 6). Across all users in the study, question 12 was altered a statistically significant amount more than the rest (*z*=3.054, *P*=.0023). Question 12 belongs to the wearable domain and relates to the number of NikeFuel points earned. It is assumed that users frequently updated this amount, more than the others, because of the variability in the data generated from the wearable device each day when they were active.

**Figure 7:**
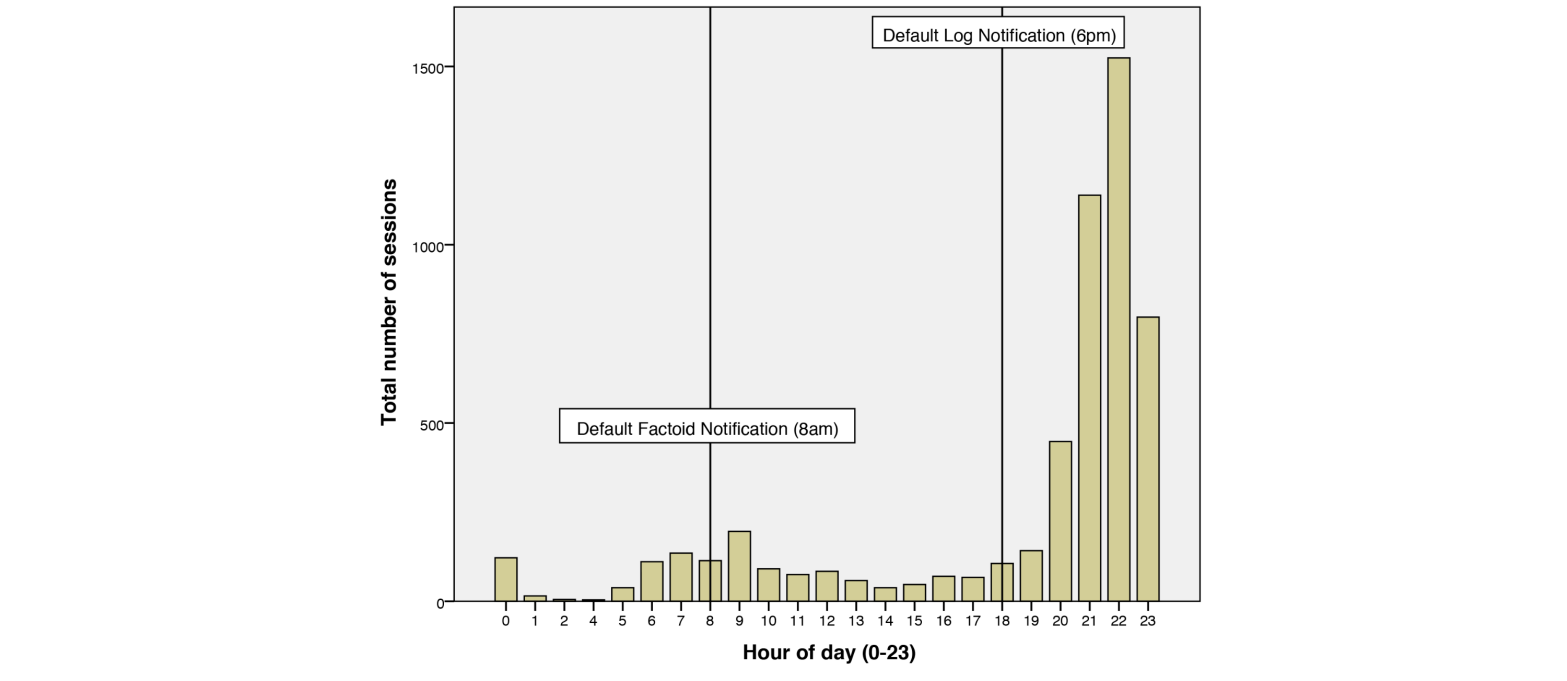
Bar graph showing the typical hours of use, with morning activity around the default daily fact notification time at 8 AM and activity peaking at 10 PM, 4 hours after the default log notification time.

The app was distributed with two default notification times. The first notification was issued in the morning at 8 am by default, which reminded the users to check their daily fact every day. The second notification was issued at 6 pm by default, which reminded the user to complete the questions in the app’s log tab. Analysis of app usage times (Figure 7) shows that the users do have a period in the morning around 7-9 am that they use the app. In the evening, however, app usage rapidly increases around 8 pm and declines sharply after 11 pm. It is believed that the users wait until the end of the day before entering their log data, so that it is the most valid representation of their day. This behavior may also be encouraged by the fact that users cannot alter their previous day’s log once the day has passed.

### User Survey

Upon the close of the study, an exit survey was issued to those in the treatment group. A total of 41 participants completed the survey. The survey acted to gather users’ motivations for behavior change and thoughts on the various components of the study, how they used them, and where they felt improvements could be made. First, users were asked how often they used the app (Table 2).

**Table 2:** Respondents’ answers to survey question: “Over the six month Gray Matters intervention period (April 2014 – October 2014), how often did you use the App?”

Usage	N	Mean	Standard deviation
Months used	39	5.54	1.315
Days per week	38	6.21	1.695
Times per day	38	1.66	3.122

#### Motivations

In addition, the survey acted to glean how the app altered motivations toward various parts of the intervention. The survey also revealed that the app motivated users to perform physical activity (never: 14.6%, rarely: 12.2%, sometimes: 24.4%, often: 31.7%, and all of the time: 17.1%) and make healthier food choices (never: 12.5%, rarely: 2.5%, sometimes: 17.1%, often: 48.8%, and all of the time: 17.1%). When queried about their past, current, and future behaviors, 46.3% said they definitely would continue with their physical activity changes and 31.7% wanted to continue and increase their activity; 46.3% wished to continue their improved eating habits, with 29.3% wanting to continue and improve. When asked if they would continue using the app, 46.3% said they would not, 29.3% said they likely would not, and 24.4% said they would continue.

#### Future App Feature Elicitation

In addition, users were asked about features that they wished were included in the app. A total of 68.3% of users wished that guidelines were based on their “current” health status, 34.1% wished they could set their own target goals, 53.7% wished they could focus the daily facts on specific behavior goals of interest, and 51.2% wished to receive text feedback if they had made good progress or no progress. A total of 53.7% wished they could compare their behaviors with others relative to their age, gender, and initial fitness status. Regarding the wearable device and app interaction, 70.7% of poststudy survey respondents wished that their wearable device automatically synched to the app. Such a feature would greatly reduce user burden of data entry.

### App Usage and Clinical Outcomes

During the duration of the study, 122,719 behavioral logs were uploaded to the central database. These logs have been analyzed for trends and correlations with clinical and biological markers recorded at the beginning and end of the intervention.

#### Number of Times App Used per Week

Logically, it is hypothesized that increased exposure to the app and its material would result in favorable outcomes, both in behavior change and in clinical markers. First, the number of times that the app was launched per week was calculated and categorized into groups (<1, 1-3, 3-5, 5-7, 7+ per week). These groups were then evaluated with various clinical and biometric measurements taken from the participants at the start and end of the study, along with the control group.

**Figure 8:**
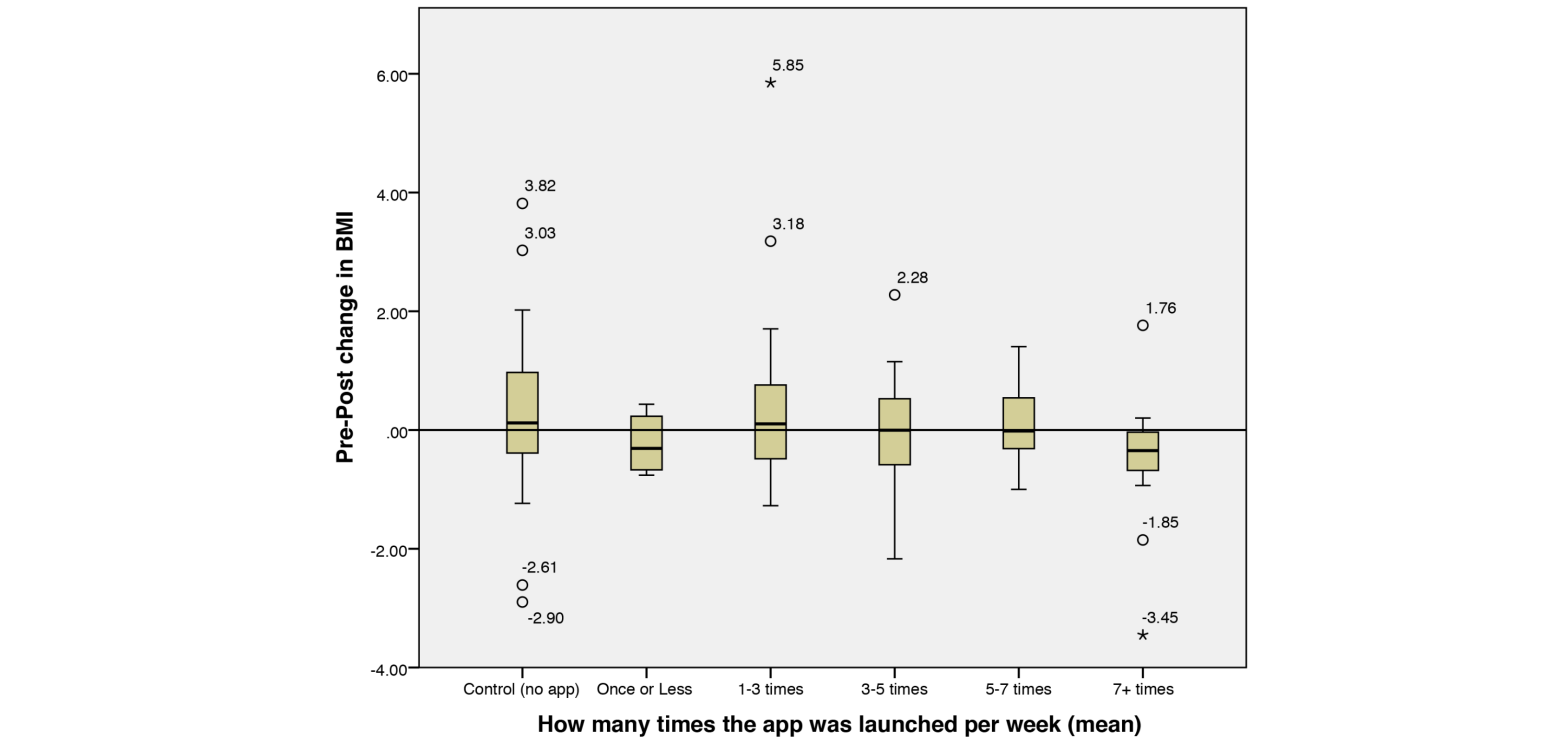
Boxplot showing no app (control) and grouped app launches per week (treatment) against observed changes in body mass index (BMI). Outliers are plotted as individual points.

**Figure 9:**
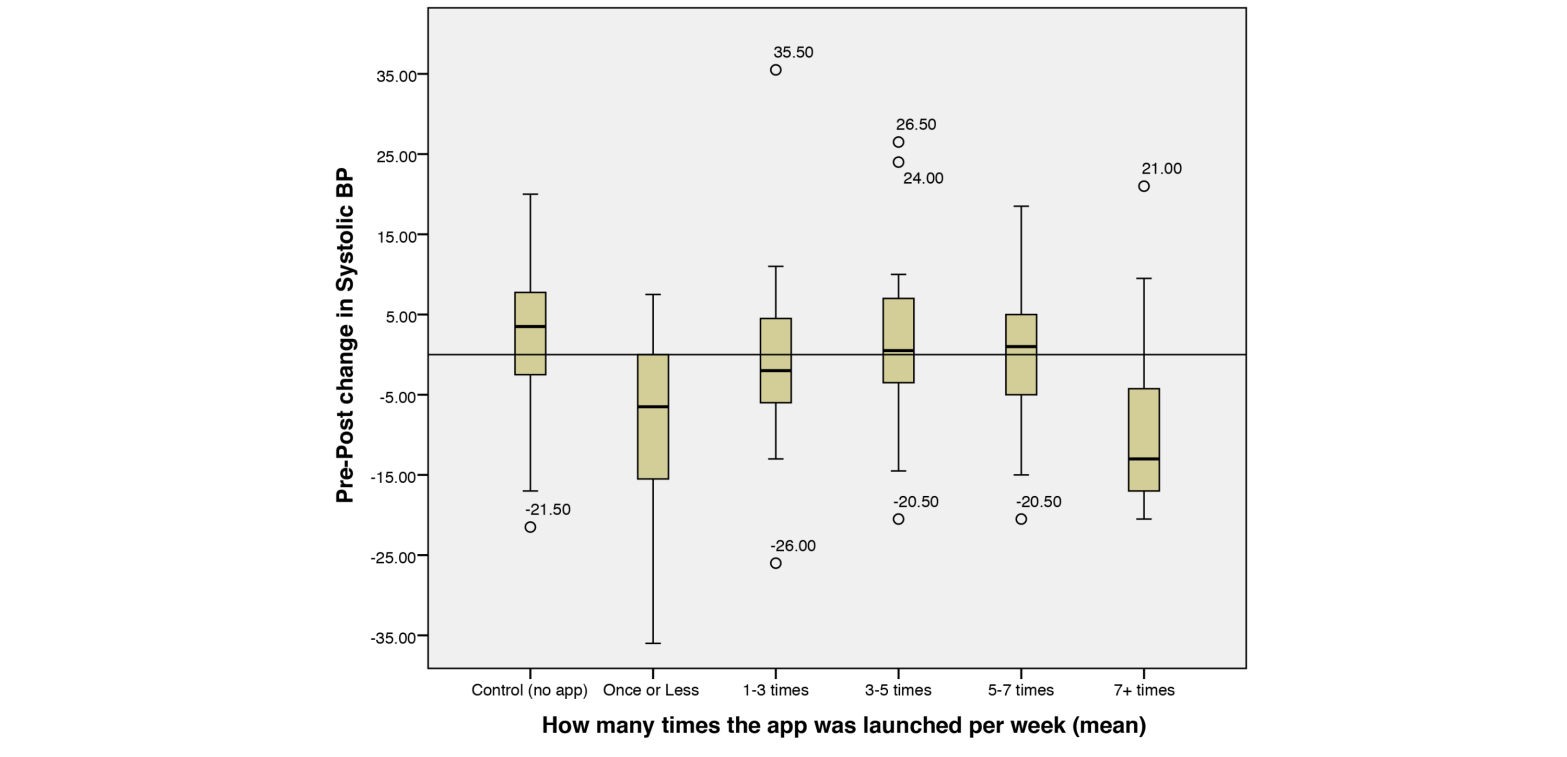
Boxplot showing no app (control) and grouped app launches per week (treatment) against observed changes in systolic blood pressure (BP; mm Hg). Outliers are plotted as individual points.

From a high level, it is evident that increased app exposure had an observable effect on various clinical measurements, in particular for BMI (Figure 8) and systolic blood pressure (SBP; Figure 9).

It can be seen that the control group had undesirable increases over the intervention period, whereas the treatment group had sustained or reduced the measurements. Notably, those who looked at the app *more than 7* times per week appear to have the largest reduction in BMI and blood pressure, whereas those who looked *less than 7* times and *more than 1* vary in their results. It is also interesting to note that those who looked at the app *once or less* per week also maintained favorable rates of decline. It is proposed that these users are self-sufficient in their efforts to effect behavior change and do not require the app to aid them. To further investigate the app’s apparent effect, we analyzed various functions of the app in relation to clinical outcomes.

#### Compliance to Log Entry and Clinical Observations

The average number of logs completed per day was analyzed for correlations to the clinical changes observed in the study, suggesting the following hypothesis:

H_0_: There is no supported relationship between daily log and clinical or biological markers.

H_1_: The number of logs completed each day will correspond to greater change in clinical and biological markers.

##### Continuous Variables

Analysis shows that daily log completion rates show no relationship between pre-post BMI scores (*r*=.016, *P*=.872) and diastolic blood pressure (*r*=.064, *P*=.523). There is a weak positive correlation found in SBP (*r*=.28, *P*=.784) and weak negative relationships in resting heart rate (*r*=−.121, *P*=.23) and blood carotenoids (*r*=−.105, *P*=.294). Further correlation analysis was completed on the biological markers, which also showed positive, but weak, correlation between the number of logs completed and pre-post total cholesterol (*r*=.145, *P*=.91) and triglycerides (*r*=.145, *P*=.15), and negative weak correlation in serum glucose (*r*=−.88, *P*=.382) and blood insulin levels (*r*=−.105, *P*=.296). Nevertheless, calculating partial correlation, controlling for the number of days the participant had the app installed, highlighted toward a significant correlation between total cholesterol and average questions per day (*r*=.193, *P*=.055). Adding an extra control for the participant’s initial recorded total cholesterol levels resulted in a significant correlation (*r*=.228 *P*=.024). We therefore reject the null hypothesis for this particular case.

##### Dichotomous Variables

Using domain knowledge, it was possible to group the clinical and biological markers into dichotomous groups (improvement or no improvement), which allowed for further analysis to be carried out. Independent samples *t* tests showed that participants who improved their high-density lipoprotein (HDL) cholesterol levels during the study duration answered a statistically significant higher number of questions per day (mean 8.30, SD 2.29) than those with no improvement (mean 6.52, SD 3.612), *t*_97.74_=−3.051, *P*=.003.

#### Achieving Recommended Daily Targets and Clinical Observations

Participants’ self-reported behaviors were analyzed to find the frequency and percentage of times that they achieved the recommended daily goal value for each question. The following hypothesis is tested:

H_0_: There is no supported relationship between achieving recommended values and clinical or biological markers.

H_1_: The higher the number of recommended goals achieved, the greater the degree of change in clinical and biological markers.

##### Continuous Variables

Correlation analysis between a participant’s mean percentage of recommended goals achieved, across the study duration, and observed clinical measurement changes showed the following: no relationship for systolic (*r*=−.013, *P*=.896) and diastolic (*r*=−.35, *P*=.732) blood pressures and no relationship in carotenoids (*r*=−.013, *P*=.895). Negative and Positive, but weak, correlation was found in resting heart rate (*r*=−.107, *P*=.285) and also BMI change (*r*=.157, *P*=.116) respectively. Biomarker changes were also correlated against percentage of recommended values achieved showing no correlation in serum glucose (*r*=−.075, *P*=.455) and blood insulin levels (*r*=−.049, *P*=.624). Positive, but weak, correlation was found for pre-post triglyceride values (*r*=.155, *P*=.124). Significant correlation, at the 95% confidence interval, was found in pre-post total cholesterol (*r*=.217, *P*=.03). The null hypothesis is accepted for all cases except this case.

##### Dichotomous Variables

Once again, each pre-post clinical and biological reading was categorized as either improvement or no improvement. For each individual, a baseline performance level was calculated from his or her self-reported behaviors in the first week of enrollment. Because there were a number of individuals within the treatment group who were highly active and maintained a healthy lifestyle, to reduce the ceiling effect on the data the first quintile (n=20) of participants were removed from the analysis. Using the dichotomous groupings of improvement and no improvement, significant correlations were found between daily goal percentage achieved and BMI reduction (*r*=.264, *P*=.017). An independent samples *t* test showed participants who decreased their BMI performed significantly better in attaining their recommended daily goals (mean 56.21%, SD 30.4%) than those who increased their BMI (mean 40.12%, SD 29.1%), *t*_80_ = −2.449, *P*=.017. Further analysis showed that 69.2% (n=18) of those who achieved a mean performance percentage of 60% or higher, across all domains, reduced their BMI during the study, whereas 60.7% (n=34) of those who did not, increased their BMI. Analysis of cross tabulation shows that those who achieved more than 60% of their recommended daily goals were 1.762 times more likely to decrease their BMI during the study, or 0.507 times less likely to increase their BMI, than those who did not achieve 60% (Table 3).

**Table 3:** Odds ratio and relative risk analysis for participants who achieved more than 60% of their recommended daily targets (mean) and body mass index change outcome.

	Value	95% confidence interval	
Lower	Upper
Odds ratio for recommended targets achieved >60% (achieved/did not achieve)	0.288	0.107	0.774
For cohort BMI^a^ change = increased	0.507	0.274	0.936
For cohort BMI change = decreased	1.762	1.164	2.667
N of valid cases	82		

^a^ BMI: body mass index.

#### Physical Activity and Clinical Observations

Participants’ reported their levels of physical activity via 3 self-reporting questions:

1. Number of minutes performing moderate physical activity

2. Number of minutes performing vigorous physical activity

3. NikeFuel points earned via wearable device.

Each participant’s results were analyzed for correlations between these values and clinical observations. The following hypothesis is tested:

H_1_: The higher the number of minutes performing physical activity/higher the NikeFuel points, the greater the degree of change in clinical and biological markers.

H_0_: There is no supported relationship between achieving physical activity levels and clinical or biological markers.

Using the dichotomous variables (improvement or no improvement), each physical activity feature was analyzed. Again, using the baseline performance metric calculated in the first week of observation, participants in the last decile (bottom 10%) were excluded from the analysis to reduce ceiling effects. An independent samples *t* test found that the remaining participants (n=92) who decreased their BMI (n=45) reported statistically significantly more vigorous physical activity (mean 23.94, SD 10.76 minutes) than those who increased their BMI (mean 19.09, SD 12.36 minutes), *t*_90_ = 2.002, *P*=.048. Interestingly, no correlation was found with moderate physical activity levels or NikeFuel points and BMI reduction status. Conversely, upon removing the first quintile, it was uncovered that those who improved their levels of HDL cholesterol during the intervention achieved significantly higher NikeFuel points on a daily basis (mean 2569.39, SD 641.17) than those who observed no improvement (mean 2233.9, SD 800.34), *F* (82)=−2.052, *P*=.043. Literature in the area of endocrinology and metabolism supports this observation as physical exercise is associated with increases in HDL [66].

#### Stress Reduction Effort and Clinical Observations

Participants’ self-reported stress reduction efforts were analyzed for their effect on clinical measures. Participants' SBP were recorded before and after intervention and categorized into low (<90), ideal (90-120), prehypertension (120-140), and hypertension (>140). Those with nonideal SBP at their preintervention recording (n=50) were analyzed to observe if a change of category occurred during the intervention. Changes observed in these participants were categorized into 3 groups: improvement (n=13), no improvement (n=14), and deterioration (n=23). One-way analysis of variance of their category changes showed a significant correlation between efforts to reduce stress (effort rated 1-10, where 10 is high effort) and SBP category change as a whole, *P*=.035 (excluding first quintile of baseline performers). Multiple comparisons of the 3 groups showed significance between those who had no improvement (mean 3.11, SD 2.32 effort rating) and those who had deteriorated (mean 5.28, SD 2.105 effort rating), *P*=.028. No significant difference was found between improvement (mean 4.18, SD 1.89 effort rating) and the remaining groups.

#### Demographic Data Versus Percentage of Recommended Values Achieved

**Figure 10:**
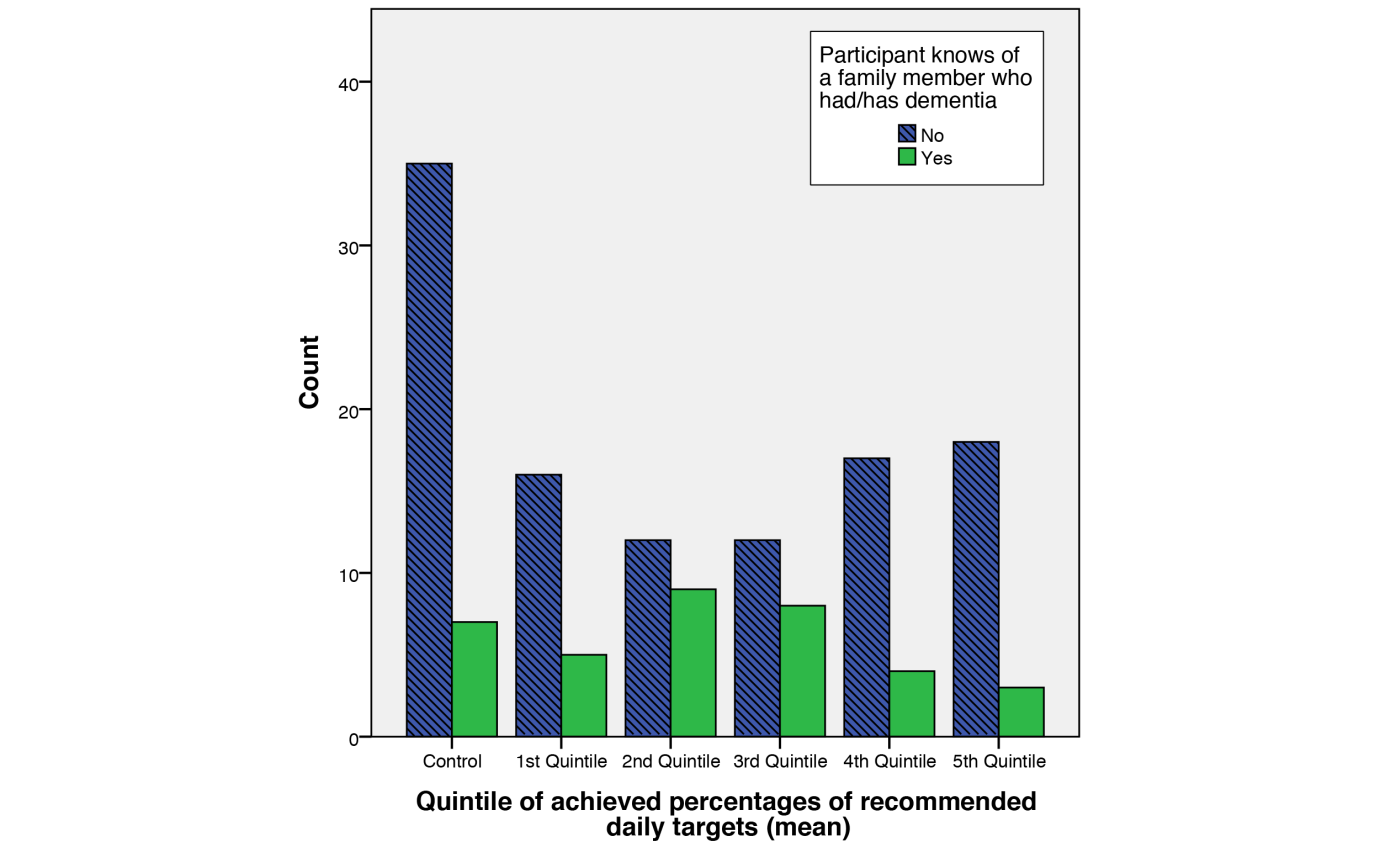
Bar chart showing the log performance quintiles against the number of users who report to have known of a family member having/had dementia.

The percentage of recommended values achieved for the entire treatment cohort was categorized into quintiles (1=highest, 5=lowest). These performance quintiles were then compared with a number of demographic variables collected at the start of the study. Analysis of these data showed relationships between a participant’s achieved percentages and whether that participant knew of a family member having dementia. This relationship is apparent between the second and fifth quintiles (Figure 10). Partial correlation within these quintiles, controlling for number of days enrolled in the study, shows significant correlation (*r*=.232, *P*=.036).

**Figure 11:**
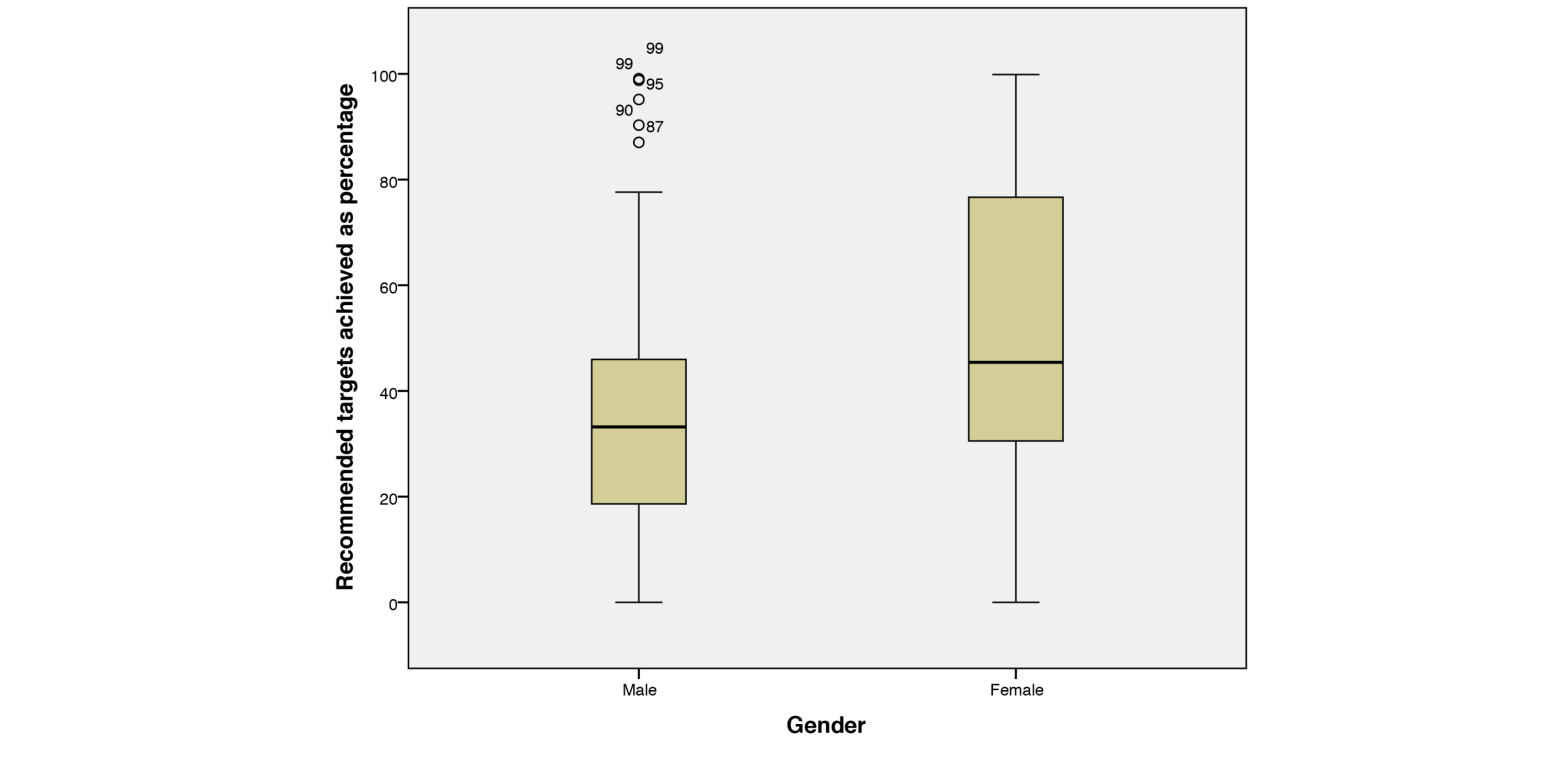
Boxplot showing distributions of male and female mean percentages of achieved targets across all domains.

Percentages achieved (0%-100%) and gender (male or female) were also analyzed (Figure 11). Independent samples *t* test shows that females achieved a statistically significant higher percentage of recommended targets (mean 52.44, SD 29.24) compared with their male counterparts (mean 38.69, SD 28.50), *t*_102_ = −2.302, *P*=.023.

It would appear that users who have family members with dementia are motivated to reach their recommended daily targets, therefore performing better, perhaps because of first-hand experience with the condition. In addition, analysis shows a visible correlation between gender and the ability to reach the recommended daily target values. The reasons behind this observation are currently unclear and require additional analysis; however, they could relate to motivations, occupation, and education level.

## Discussion

### Principal Findings

The mobile phone app provided a novel method to remotely monitor participants in a behavior change intervention, while also facilitating the delivery of intervention material. In addition, analysis of exit survey shows that the app facilitated stages 3-5 of the TTM, preparing participants for change, allowing them to accurately monitor and assess their actions, and encouraging continued maintenance and improvement of their desired behaviors. Results from the exit survey showed that most users wished to continue their behavior change efforts, which if maintained, are expected to yield superior outcomes in AD prevention.

In this trial, the recommended values for each behavior played a key role in the uniform assessment of participants’ performance. Analysis of pre-post measurements from the treatment group showed clear physiological changes in those who achieved the highest in their attempts to meet recommended values. This was especially apparent in those who were previously underachievers in certain behavioral domains, before the study (based on the first week of observed behavior logs). Effects observed included a desirable lowering of BMI, improvements in HDL and low-density lipoprotein cholesterol levels, improvements in SBP, lowering of resting heart rate, and improvements in perceived stress levels.

**Figure 12:**
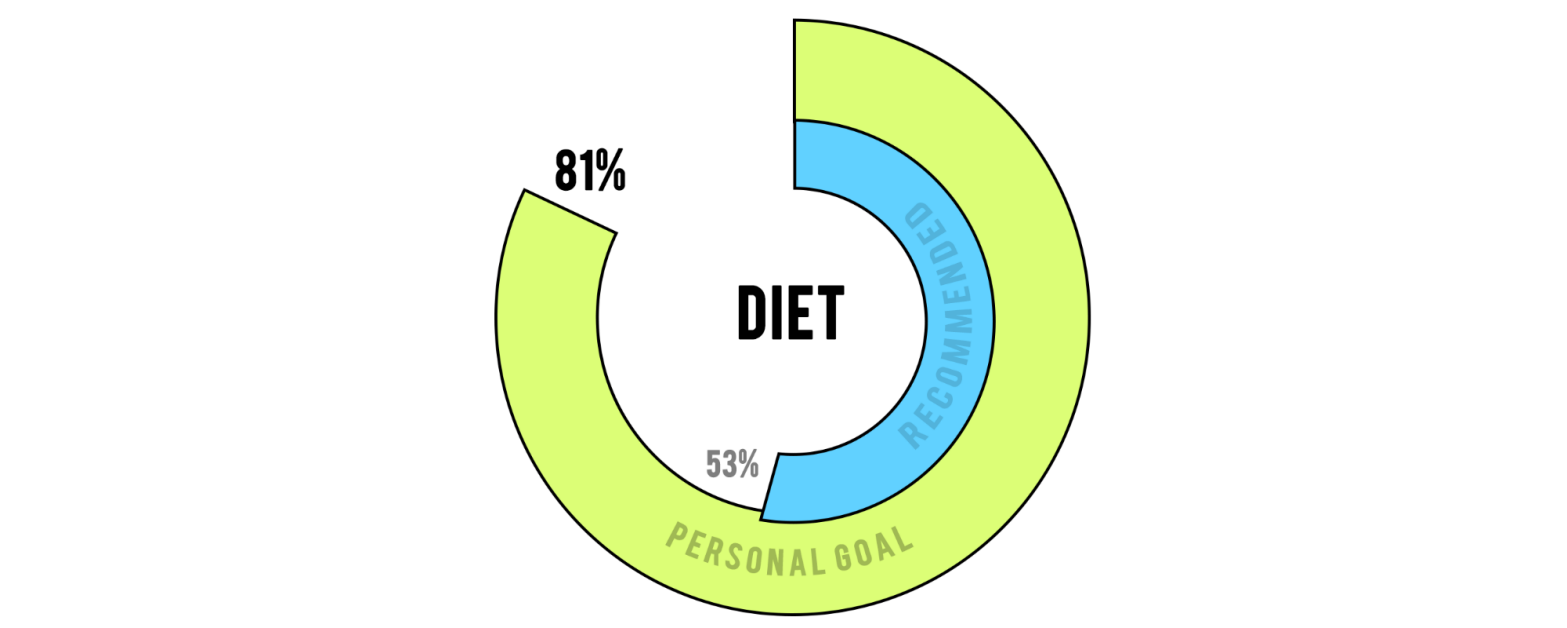
A prospective graphical representation of a user’s efforts against globally recommended values and his or her personal goals.

Regarding user experience, most app users stated that they wished to alter their recommended values to be based on their “current” health status, whereas others wished to manually set their own target goals. Such a feature could improve engagement with the app, at the detriment of a true representation of progress. A compromise would be to present the user with their efforts against both personal and global targets (Figure 12).

Half of the users wished that their educational material was focused on a specific domain of interest, rather than evenly spread throughout all behavioral domains. Such a focus may be beneficial if the user requires extensive change in one particular domain, but for the purpose of a multidomain intervention the investigators decided it was of great importance to educate across all domains.

### Limitations

The findings of the study may be biased toward the study cohort’s locale and ethnic group. The study cohort was predominantly white (96.6%) and the participants resided in a county that is classified as 96.23% rural [67]. Although desirable changes in behavior were observed within this cohort, additional research is required to examine the efficacy of the approach within other countries, in various settings, spanning numerous ethnic groups.

Within this larger study, additional work would be required to accommodate and account for the cultural, regional, and religious differences across groups; for example, adjusting dietary recommendations based on religious practice.

### Future Improvements

Through direct communications with participants and survey analysis, various aspects of the app and supplementing technology have been identified for improvements for a future version of the study.

#### Wearable Device Integration

The Nike FuelBand’s proprietary and nondisclosed metric of NikeFuel points is rather ambiguous for the purpose of a scientific study. Many users reported that the device did not accurately award them with points during activity and, conversely, awarded them with points when they were performing sedentary tasks, such as when they were driving their car. These false positives removed the opportunity to use the data to validate reported physical activity with the FuelBand’s NikeFuel metric. In agreement with the participants' comments, a recent study assessing the validity of commercially available activity monitors found the FuelBand to be one of the weakest performers overall, undercounting daily step count, on average, by 2529 steps [68]. There are now numerous commercially available alternatives that allow for greater granularity in their data, such as step counts, distance travelled, sleep quality, and resting heart rate. Many of these wearables allow for direct integration with apps via simple application program interface calls. Because of this feature, self-reported sleep and physical activity may be correlated against the data collected directly from the wearable device to examine validity. The future iteration plans to seek alternatives.

As discussed earlier, the users also had the burden of repeatedly entering their NikeFuel points via the log screen. This user burden of data entry can be greatly reduced by enabling the transfer of data from relevant wearable devices directly to the app, greatly increasing the convenience of the solution.

#### Social Network Integration

Participants had informed us that they wished that the app were more socially engaging. For future development we have identified that a social element is required, allowing users to add friends with whom they can publicly compare their efforts. Integrating the app with existing social networks, such as Facebook and Twitter, can facilitate this feature. Social network integration will allow the users to find friends already in their network, who are also using the app. From here they may compare their own accomplishments with those in their friend group, thus offering an opportunity to heighten motivations for change. In addition, integration with these networks will also allow users to post their accomplishments to their public pages, allowing those outside the study to view their efforts and provide an opportunity for additional peer support, while boosting the public profile of the study.

#### Personalization

There is a huge opportunity for personalization in all aspects of the app. Users of the Gray Matters app have suggested that they would like to set their own targets and behavior change goals. This includes adding or removing domains based on a user’s motivations. Daily fact delivery could also be revised to prioritize daily facts from a domain of interest to the user.

#### Higher Granularity Reporting

Within the study, participants were asked to report behaviors that were reasoned as favorable by the investigators because of their role in AD prevention. However, the participants were not asked to report behaviors that should be avoided. For example, although participants were encouraged to consume fruits and nuts, they were not asked to report how many refined sugars or processed foods they consumed. Using solely the measure of desirable food intake, without observing the undesirable food intake, results in a skewed representation of diet macronutrients and overall calories consumed.

#### Additional Behavioral Domains (Smoking Cessation)

Smoking cessation was not included in the original study, as there is an extremely low rate of smokers in the Cache County area [69]. Nevertheless, if the Gray Matters study were to target a larger geographical area, state or nationwide, facts and suggestions related to smoking cessation would be included.

#### Improvement of Daily Fact and Question Database

On an ongoing basis, we will strengthen and expand the daily fact database, adding new facts and suggestions, with each vetted using a modification of the rating system developed by the Grading of Recommendations Assessment, Development and Evaluation working group [70]. Analysis of in-app behaviors showed that users had tapped on questions numerous times to help them understand the exact semantics of a question. In addition, some external feedback outside of the study cohort suggested that some of the daily facts could have been clarified. As such, in future versions resources should be allocated to analyze the average user’s interpretation of daily facts and questions, to ensure that confusion is limited.

#### Distribution

A number of suggestions were provided by users of the app informally via email during the duration of the study. A familiar complaint included improving the distribution method of the app. The TestFlight platform, although useful for maintaining control of distribution, was developed for tech-savvy users, not for clinical interventions. As such, many users had problems registering with the platform and subsequently approving certificates and downloading the app. In the next iteration, all distribution will take place via the platform’s official app repositories, iOS App Store and Google Play Store.

### Conclusions

The prevailing theme of this paper has been to express the benefit of using a mobile phone app as a core component of a behavior change intervention—to yield the advantages offered by the pervasive nature of the mobile phone within an individual’s daily life and routines. In this study, the mobile phone offered the opportunity for clinical effect to occur through behavior change. The app excelled as a delivery platform for the intervention, enabling the dissemination of educational intervention material, while simultaneously monitoring and encouraging positive behavior change. Although the effect of behavior change in midlife, observed during the 6-month RCT, on future AD risk is still relatively unclear, it is evident that participants in the treatment group had favorable improvements across numerous physiological domains, suggesting that a sustained effort would yield superior outcomes in the future.

## Abbreviations

AD: Alzheimer disease
BMI: body mass index
CDC: Centers for Disease Control and Prevention
HBM: Health Belief Model
HDL: high-density lipoprotein
RCT: randomized controlled trial
SBP: systolic blood pressure
SMS: short message service
TPB: Theory of Planned Behavior
TTM: Transtheoretical Model

## Appendix

Multimedia Appendix 1 CONSORT-EHEALTH Checklist (v1.6.1).
